# Treatment of Popliteal Pterygium Using an Ilizarov External Fixator

**DOI:** 10.4055/cios.2009.1.4.236

**Published:** 2009-11-25

**Authors:** Hyoung-Min Kim, Il-Jung Park, Changhoon Jeong

**Affiliations:** Department of Orthopedic Surgery, The Catholic University of Korea, Bucheon St. Mary's Hospital, Bucheon, Korea.

**Keywords:** Popliteal pterygium syndrome, External fixator, Ilizarov

## Abstract

Popliteal pterygium syndrome is a rare congenital disorder that consists of popliteal webs and craniofacial, genitourinary and extremity anomalies. Only moderate successful surgical excision of the fibrotic band within the popliteal web has been reported because the nerves and vessels in the affected site are short and displaced into the web and they are attached to adjacent tissues. We performed hamstring tenotomy on the ischial tuberosity, tenotomy of the flexor hallucis longus and Z-lengthening of the Achilles tendon on the ankle in our patient, and this was followed by gradual correction using an Ilizarov external fixator. Full extension of the knee joint was achieved at the ninth postoperative week. However, some recurrence of flexion contracture was noted at two years follow-up. Gradual soft tissue lengthening with an Ilizarov external fixator can be one of the optimal procedures when excision of a fibrous band and Z-plasty are not possible due to severe adhesion of the nerves and vessels into a fibrotic band. However, a cautious approach is recommended when considering the high risk of recurrence.

Popliteal pterygium syndrome (PPS) is a rare disorder that's characterized by flexion contractures of the knee and popliteal webbing. The cause of the PPS has not yet been firmly established. This disorder sometimes affect other joints and it is often combined with craniofacial, oral cavity and genitourinary anomalies.^1^,2) PPS requires aggressive treatment not only for functional reasons, but also for cosmetic and hygienic reasons. One of the most recommendable procedures is lengthening of the skin and soft tissues by excision of fibrous bands and Z-plasty at an early stage. However, complete lengthening is not often achievable because the nerves and vessels in the affected site are short and displaced into the web and attached to adjacent tissues. In such cases, nerve grafting is additionally carried out following soft tissue lengthening. Sometimes femoral extension osteotomy or femoral shortening or knee arthrodesis is concomitantly performed. In some severe cases, amputation becomes an option.^1^-5) We report here on a case of PPS, and the patient was treated with an Ilizarov external fixator for flexion contracture of the knee joint and the equinus deformity of the ankle joint following unsuccessful excision of fibrous bands and soft tissue lengthening.

## CASE REPORT

**Fig. 1:**
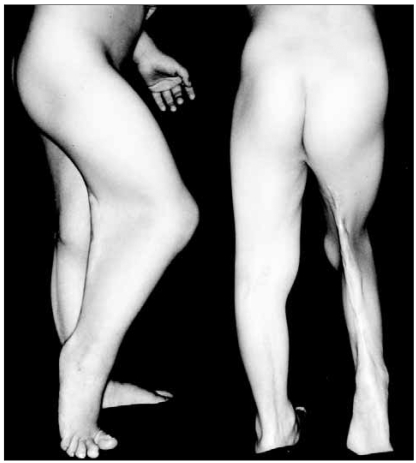
Photographs showing the flexion contracture and web on the popliteal space of the right knee joint.

A 7-year-old male was admitted to our hospital for flexion contracture of the right knee and popliteal pterygium. The combined anomalies included cleft lip, cleft palate, congenital lip sinus, mucus membrane adhesion (the upper and lower buccal mucosa to the tongue), atrophy of the scrotal wall, small testes and a small penis. Bilateral popliteal pterygium was found at the 2nd postnatal month. Lengthening of the skin and soft tissues by excising the fibrous band and Z-plasty were performed for the left pterygium. However, only Z-plasty was done for the skin and soft tissue lengthening for the right pterygium because excision of the fibrous band was not possible due to severe adhesion of the sciatic nerve to the fibrotic band to the sciatic nerve. Thereafter, soft tissue lengthening was attempted in another hospital at the age of 5 because the flexion contracture and popliteal pterygium of the right knee relapsed, but the adhesion to the sciatic nerve also caused a failure. On the physical examination, 45° of knee flexion contracture and 30° of plantar flexion contracture were observed (Fig. 1). Femoral arteriography revealed no significant displacement of the popliteal artery. Plain radiography showed that the femur and the tibia were normal, but the calcaneus was hypoplastic.

**Fig. 2:**
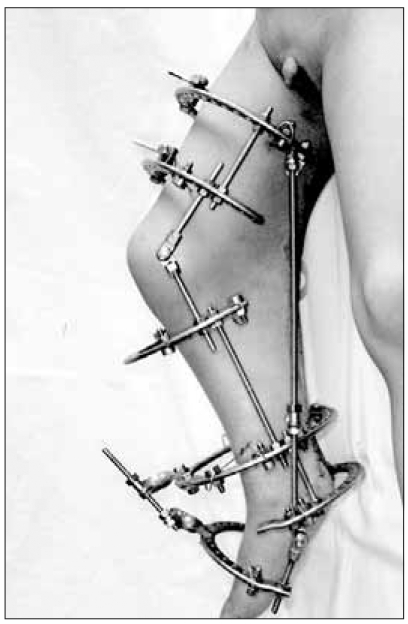
Photographs showing the application of the Ilizarov external fixator.

**Fig. 3:**
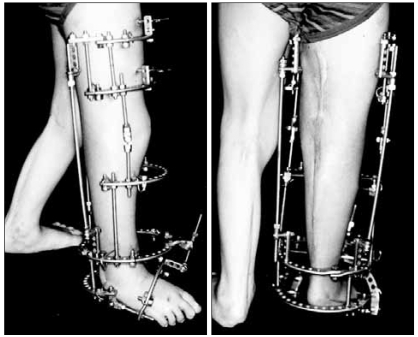
Photographs showing correction of the flexion contracture of the knee joint and ankle joint at 9 weeks after the operation.

We performed hamstring tenotomy on the ischial tuberosity, tenotomy of the flexor hallucis longus and Z-lengthening of the Achilles tendon on the ankle, and this was followed by the application of an Ilizarov external fixator (Fig. 2). The patient underwent gradual lengthening from the sixth postoperative day. At the ninth postoperative week, full extension of the knee joint and a neutral position of the ankle joint were achieved (Fig. 3). At the sixteenth postoperative week, the Ilizarov external fixator was removed and a knee-ankle-foot orthosis was applied. During the two years of postoperative follow-up, 15° of knee flexion contracture and 20° of plantar flexion contracture recurred.

## DISCUSSION

PPS is a rare genetic condition that's characterized by popliteal webbing, craniofacial anomalies, genitourinary anomalies and skeletal anomalies. The same as for Van der Woude syndrome, mutation in the interferon regulatory factor 6 gene is presumed to be responsible for the disorder.6) Bartsocas and Papas7) postulated that PPS can be inherited in an autosomal dominant and an autosomal recessive manner; autosomal dominant PPS is more common and it shows variable penetrance, but autosomal recessive PPS occurs in patients who are born to unaffected parents and it is associated with more severe anomalies and mental retardation. Yet according to the recent report of Giannotti et al.,8) PPS is an autosomal dominant disorder that's caused by interferon regulatory factor 6 gene mutations. With regard to the autosomal recessive forms described by Bartsocas and Papas,7) Giannotti et al.8) postulated that those cases should have been diagnosed as another disorder with symptoms that were similar but more severe than those of PPS. The patient and all the other affected siblings described in the report by Bartsocas and Papas7) presented with more severe anomalies and they died within the first nine weeks of life. We believe that the case described in this report should be diagnosed as PPS rather than Bartsocas-Papas syndrome.

PPS can be diagnosed based on the presence of cleft lip, cleft palate, genital hypoplasia and popliteal webbing, but care should be taken to differentiate it from multiple pterygium syndrome, arthrogryposis multiplex congenita, sacral agenesis and myelomenigocele. Multiple pterygium syndrome is characterized by pterygia of several joints such as the axilla, the volar side of the elbow and the interphalangeal areas, and patients with multiple pterygium present with other various deformities.2) Arthrogryposis multiplex congenita is usually symmetrical and it involves the extremities and it significantly limits the joint movement due to combined amyotrophy and it causes the loss of skin creases. The case in our report could be diff erentiated from arthrogryposis multiplex congenita because the patient had no amyotrophy or phalangeal deformities. Sacral agenesis and myelomeningocele could also be ruled out because no vertebral deformation or laminar defects were noted on the plain radiography.

There are various treatment methods for PPS. Surgical treatment is preferred because the results of conservative treatment, including serial casting or traction, have been unsatisfactory. Among these surgical treatments, lengthening of the soft tissues such as the skin, muscles and ligaments using resection of fibrous bands and Z-plasty was the most preferred technique.^1^-3) However, this technique is not applicable to many cases where the sciatic nerve is displaced into the webbing and it is attached to the fibrous tissues. In that circumstance, nerve grafting is additionally employed following soft tissue lengthening. Sometimes femoral extension osteotomy, femoral shortening or knee arthrodesis is concomitantly performed. In severe cases, amputation also becomes an option.^1^-5) Oppenheim et al.4) reported that excision of fibrous bands and Z-plasty were possible in only 2 of 7 cases of PPS (4 patients) and the rest of the cases required secondary operations such as femoral extension osteotomy, femoral shortening or amputation due to severe adhesion to the sciatic nerve. The patient described in our report had bilateral popliteal pterygium and the left one was corrected with Z-plasty, while the right one was untreatable even with a couple of operations. Correction of deformities might be obtained with femoral extension osteotomy, femoral shortening or knee arthrodesis. However, considering the patient's age and the prospects of severe shortening of the lower extremity and the leg length discrepancy, we performed hamstring release at the ischial tuberosity, tenotomy of the flexor hallucis longus and Achilles tendon Z-lengthening on the ankle, and this was all followed by gradual lengthening using an Ilizarov external fixator.

An Ilizarov external fixator is commonly used for correction of shortening or deformity, and it is also employed for the treatment of joint contractures. According to Gillen et al.,9) the device allowed all 4 of their patients with PPS to obtain 0° of knee extension, but 15-30° of contracture recurred postoperatively. This is rebound phenomenon is a major obstacle in the treatment of PPS. This recurred contracture is known to appear following almost every treatment for PPS, albeit to a different degree. The patient in our report could obtain 0° of knee extension via gradual soft tissue lengthening with using an Ilizarov external fixator and the patient wore a knee-ankle-foot orthosis postoperatively to prevent relapse. Some authors have recommended the use of an orthosis following surgery based on their observations.^9^,10) However, we believe that the efficacy of an orthosis should be assessed in future studies when considering that it did not result in preventing relapse. Gillen et al.9) reported that gradual soft tissue lengthening using an Ilizarov external fixator could lead to complications such as subluxation or dislocation of the knee, fracture or temporary loss of sensation. Fortunately, other than the pin tract infection that was treated with oral antibiotics, no complications were noted in our patient.

Gradual soft tissue lengthening with an Ilizarov external fixator can be one of the optimal procedures when excision of fibrous band and Z-plasty are not possible due to adhesion to the nerves or blood vessels in the popliteal pterygium. However, a cautious approach is recommended when considering the high risk of recurrence.

## References

[1] Herold HZ, Shmueli G, Baruchin AM (1986). Popliteal pterygium syndrome. Clin Orthop Relat Res.

[2] McCall RE, Budden J (1992). Treatment of multiple pterygium syndrome. Orthopedics.

[3] Herzenberg JE, Davis JR, Paley D, Bhave A (1994). Mechanical distraction for treatment of severe knee flexion contractures. Clin Orthop Relat Res.

[4] Oppenheim WL, Larson KR, McNabb MB, Smith CF, Setoguchi Y (1990). Popliteal pterygium syndrome: an orthopaedic perspective. J Pediatr Orthop.

[5] Saleh M, Gibson MF, Sharrard WJ (1989). Femoral shortening in correction of congenital knee flexion deformity with popliteal webbing. J Pediatr Orthop.

[6] Kondo S, Schutte BC, Richardson RJ (2002). Mutations in IRF6 cause Van der Woude and popliteal pterygium syndromes. Nat Genet.

[7] Bartsocas CS, Papas CV (1972). Popliteal pterygium syndrome: evidence for a severe autosomal recessive form. J Med Genet.

[8] Giannotti A, Digilio MC, Standoli L, Zama M, Dallapiccola B (1992). New case of Bartsocas-Papas syndrome surviving at 20 months. Am J Med Genet.

[9] Gillen JA, Walker JL, Burgess RC, Stevens DB (1996). Use of Ilizarov external fixator to treat joint pterygia. J Pediatr Orthop.

[10] Parikh SN, Crawford AH, Do TT, Roy DR (2004). Popliteal pterygium syndrome: implications for orthopaedic management. J Pediatr Orthop B.

